# Predictors of MRI-estimated brain iron deposition in dementia and Parkinson's disease-associated subcortical regions: Genetic and observational analysis in UK Biobank

**DOI:** 10.1177/13872877251375432

**Published:** 2025-09-15

**Authors:** Francesco Casanova, Qu Tian, Daniel S Williamson, Mitchell R Lucas, David Zweibaum, Jun Ding, Janice L Atkins, David Melzer, Luigi Ferrucci, Luke C Pilling

**Affiliations:** 1 Department of Clinical and Biomedical Sciences, University of Exeter, Exeter, UK; 2 Translational Gerontology Branch Longitudinal Studies Section, National Institute on Aging, Baltimore, MD, USA; 3 Department of Health and Care Professions, University of Exeter, Exeter, UK

**Keywords:** Alzheimer's disease, brain, dementia, iron, neurodegeneration, Parkinson's disease, risk factors

## Abstract

**Background:**

Brain iron in specific subcortical regions increases risk of dementia and Parkinson's disease (PD). Genetic and environmental factors affect iron deposition, but underlying mechanisms are unclear.

**Objective:**

Identify risk factors and diseases associated with brain iron; assess causality using genetics.

**Methods:**

41,581 UK Biobank participants had MRI-estimated brain iron (QSM method) in five dementia or PD-associated subcortical regions (caudate, hippocampus, putamen, substantia nigra, thalamus). We investigated common risk factors (including adiposity, blood pressure, health behaviors, inflammation) and diseases observationally, using covariate-adjusted regression models, and genetically, with Mendelian randomization.

**Results:**

Participants diagnosed with Alzheimer's disease, PD, or other diseases had higher MRI-estimated brain iron. Anemia, osteoporosis, and hyperparathyroidism were associated with lower brain iron. Higher body mass index and blood pressure, smoking history, and self-reported meat consumption, increased brain iron. Hematological parameters, inflammatory and kidney biomarkers, and calcium, were also associated. Genetics support causal effects of depression, type-2 diabetes, and 7 other diseases with increased iron, but not Alzheimer's disease. Evidence supports a causal effect of osteoporosis on lower iron in the substantia nigra. We found causal associations between adiposity and proteins (including IL-6 receptor and transferrin receptor) on subcortical brain iron.

**Conclusions:**

We identified causal effects for liability to type-2 diabetes, depression, and other conditions, on subcortical MRI-estimated brain iron, but not to Alzheimer's disease, supportive of dementia as a consequence of brain iron deposition, not a cause. The role of adiposity reducing interventions on brain iron should be investigated. Relationships between brain iron, osteoporosis, calcium, and hyperparathyroidism warrant further investigation.

## Introduction

Brain iron deposition is thought to increase risk of neurodegenerative disease,^1^ yet mechanisms are complex, with interactions between chronic diseases, hepcidin production, iron retention by macrophages, and iron absorption in the gut.^2^ We previously reported that higher plasma iron and MRI-estimated iron deposition in specific subcortical brain regions are causally associated with increased risk of non-Alzheimer's dementia and Parkinson's disease (PD).^3,4^ Brain iron deposition is present in Alzheimer's disease (AD) and is associated with cognition, but may not be causal, with as yet no conclusive evidence supporting intervention with iron chelators.^5^ The underlying risk factors that increase brain iron deposition are not fully understood, with uncertainty over the importance of reported factors in the causal chain leading to disease.

Brain iron naturally accumulates over the lifecourse.^1^ The role of genetics in brain iron accumulation is supported by genome-wide association studies (GWAS)^6^ and studies of the iron-overload disease hemochromatosis.^7^ Type-2 diabetes and glycemic traits increase brain iron,^8–10^ as well as body mass index (BMI)^11^ and cardiovascular risk factors,^12^ implicating metabolic dysfunction as a driver of iron overload. Modifiable risk factors such as smoking^8,11^ and blood pressure^13^ highlight vascular mechanisms. Evidence for environmental and behavioral factors are not consistent across studies and their causal role remains unclear.^6^

The importance of establishing causality is shown by alcohol consumption: observationally, higher consumption is associated with higher brain iron, but Mendelian randomization (MR) analysis does not consistently support a causal effect.^14^ MR uses genetic variants as instrumental variables (proxies) for an exposure, reducing bias from reverse causation and unmeasured confounders.^15^

Our recent MR analysis supports a causal effect of iron in the thalamus on non-Alzheimer's dementia risk, and iron in the substantia nigra, caudate and putamen on PD risk.^3,4^ In this study we aimed to identify factors causally associated with MRI-estimated brain iron deposition (using the established quantitative susceptibility mapping (QSM) method^6^) in these 4 regions, as well as in the hippocampus because of its known role in the development of AD, using observational and genetic analyses.

## Methods

### Study sample

UK Biobank (UKB) is a cohort study of ∼502,000 UK adults aged 40–70 years at baseline assessment (2006–2010). Data includes characteristics, biomarkers, genetics, and medical records. Data are available following application (www.ukbiobank.ac.uk). The Northwest Multi-Center Research Ethics Committee approved the collection and use of UKB data (Research Ethics Committee reference 11/NW/0382). Participants gave informed consent for the use of their data for health-related research purposes. Access to UKB was granted under Application Number 83534.

### MRI iron estimation

MRI data was processed centrally and imaging-derived phenotypes (IDPs) made available to analysts. We used brain iron levels estimated by the QSM method,^6^ available in all 5 subcortical regions of interest (caudate, hippocampus, putamen, substantia nigra, and thalamus; UKB fields 24469, 24470, 24475, 24476, 24471, 24472, 24481, 24482, 24467, 24468). Mean values for right and left hemisphere measures were used. Data was available in 41,581 participants (November-2024).

### Participant characteristics, risk factors, and long-term conditions

Phenotypes from MRI assessment were age (field = 53), sex (field = 31), assessment center (field = 54), education/qualifications (field = 6138), smoking status (field = 20116), alcohol intake frequency (field = 1558), and “number of days per week of moderate physical activity 10+ minutes” (field = 884). Some groups were combined, e.g., “secondary school” (CSEs + GCSEs) qualifications, and “4+ days per week” for moderate activity. We created a combined “number of days per week of red or processed meat consumption” phenotype using four food frequency questionnaire fields: lamb/mutton (field = 1379), pork (1389), beef (1369), and processed meat (1349).

Baseline fields used were “self-reported ethnic background” (field = 21000, with subgroups combined into “White,” “Asian,” “Black,” “Mixed,” and “Other” groups), and biochemistry (n = 30 fields from UKB category = 17518) and hematology (n = 31 fields from category = 100081) fields.

We ascertained the date of first diagnosis of 88 long-term conditions from hospital episode statistics (HES, censored 30-October-2022), death certificates (censored 30-November-2022), cancer registry (censored 31-December-2020), and primary care (available in ∼45% of the cohort, censored 31-May-2016 – provider dependent). The conditions comprised 83 from the GEMINI analysis of common long-term conditions^16^ plus hemochromatosis and 4 dementia phenotype (all-cause dementia, AD, non-Alzheimer's dementia, and vascular dementia). See Supplemental Table 1 for diagnostic codes and R package `ukbrapR` v0.2.8 for code to ascertain diagnoses in the UKB Research Analysis Platform (https://github.com/lcpilling/ukbrapR/).

### Olink proteomics

Plasma proteins were measured by the Pharma Proteomics Project (UKB-PPP) using an antibody-based method (Olink Explore 3072 PEA) capturing 2923 unique proteins in 54,219 participants. This included 46,595 randomly selected participants, 6376 selected by UKB-PPP (to enrich for specific diseases), and 1268 from the COVID-19 repeat-imaging study. Detailed methods are published.^17^ There were 5222 participants with baseline proteins and QSM data.

### Genotype data

Participants were genotyped using two similar (>95% shared variants, n = 805,426) microarray platforms: the Affymetrix Axiom UK Biobank (n = 438,427 participants) and Affymetrix UKBiLEVE (n = 49,950) arrays. UKB performed genotype imputation in 487,442 participants using the Haplotype Reference Consortium and UK10K reference panels (n=∼96 million variants).^18^

### Genetic instrumental variables

For brain iron we used our published GWAS.^3^ We used published consortia meta-analysis GWAS for AD^19^ and PD,^20^ and results from GEMINI for 72 long-term conditions.^16^ For biochemistry and hematology phenotypes we used the largest available GWAS.^21^ See Supplemental Table 2 for list.

For each protein (n = 2923) we used the strongest genetic variant mapped to the gene coding for the assayed protein (the “cis-MR” variant) reported by UKB-PPP*.*^17^ Of the 2923 analyzed, 2060 had a genome wide significant (p < 5*10^−8^) cis variant. We excluded 68 with low minor allele frequency (MAF < 0.1%) in the MRI sample, and 42 on the X chromosome (missing in many outcome GWAS). This left 2018 proteins for cis-MR analysis.

We used a recent GWAS of plasma transferrin saturation (TSAT),^22^ the best marker for plasma iron content, to investigate specificity.

### Statistical analysis

R v4.2.3 was used for all analyses.

#### Observational analyses

We used linear regression models throughout, unless otherwise specified. QSM brain iron estimates were inverse rank transformed to ensure Gaussian distributions and standardized units. All models were adjusted for age at MRI assessment, sex, and MRI assessment center. Additional adjustments are detailed in the Results (e.g., age at baseline assessment for biochemistry analysis). Benjamini-Hochberg adjustment for multiple statistical testing was performed within analysis groups. Syntax is available https://github.com/AGEexeter/paper-brain_iron_causes.

We used Fisher's Z to compare estimates between two models using the following equation:

$$Z=\frac{\beta_{1}-\;\beta_{2}}{\sqrt{SE\beta_{1}^{2}+SE\beta_{2}^{2}}}$$

where $\beta_{1}$ is the estimate from model 1 and $\beta_{2}$ is the estimate from model 2, and $SE\beta_{1}$ is the standard error for $\beta_{1}$ and $SE\beta_{2}$ is the standard error for $\beta_{2}$ .

#### Mendelian randomization

For each exposure we used associated genetic variants as instrumental variables and identified associations with brain iron (QSM) using the above published GWAS. Multi-allelic SNPs were excluded. The main MR estimate is from inverse-variance weighted (IVW) analysis, which assumes there is no unbalanced horizontal pleiotropy. Additional analyses are performed to test MR assumptions, including: weighted median (assumes <50% of the weight in the analysis comes from invalid instruments); MR-Egger (assumes the genetic variants’ effect is not correlated with any pleiotropic effect on the outcome); MR-Egger (intercept term) unlike IVW the MR-Egger incept is not fixed at zero, therefore deviation from the null indicates pleiotropy. We used R packages MRlap^23^ (v0.0.3.3) and TwoSampleMR (v0.6.6) for MR analysis. MRlap performs the IVW analysis, adjusting for sample overlap and weak instrument bias (providing “corrected” betas and standard errors). TwoSampleMR performed sensitivity analyses (e.g., leave-one-out to investigate outliers). Full results available https://github.com/AGEexeter/paper-brain_iron_causes.

## Results

We used data from 41,581 UK Biobank (UKB) participants with MRI-estimated brain iron (QSM method) available at the time of analysis (November 2024). The mean age was 64·2 years (SD 7·74); 53% were female (Table 1).

**Table 1.** UK Biobank participant characteristics.

		Overall
		(N = 41,581)
Age at assessment	Mean (SD)	64.2 (7.74)
	Median [Min, Max]	65.0 [45.0, 83.0]
Sex	Female	22,050 (53.0%)
	Male	19,531 (47.0%)
Ethnicity	White	40,252 (96.8%)
	Mixed	195 (0.5%)
	Asian	541 (1.3%)
	Black	262 (0.6%)
	Other	212 (0.5%)
	Missing	119 (0.3%)
Highest qualification	Primary school	2616 (6.3%)
	Secondary school	9561 (23.0%)
	Higher qualification	28,977 (69.7%)
	Missing	427 (1.0%)
Smoking status	Never	25,859 (62.2%)
	Previous	13,933 (33.5%)
	Current	1357 (3.3%)
	Missing	432 (1.0%)
Body mass index (BMI)	Mean (SD)	26.5 (4.36)
	Median [Min, Max]	25.9 [13.4, 58.0]
	Missing	1182 (2.8%)
BMI (categories)	Normal	16,088 (38.7%)
	Overweight	16,695 (40.2%)
	Obese	7314 (17.6%)
	Underweight	302 (0.7%)
	Missing	1182 (2.8%)
Waist circumference (cm)	Mean (SD)	88.3 (12.6)
	Median [Min, Max]	88.0 [53.0, 152]
	Missing	1076 (2.6%)
Waist:Hip Ratio	Mean (SD)	0.875 (0.0883)
	Median [Min, Max]	0.878 [0.534, 1.40]
	Missing	1077 (2.6%)
Waist:Hip Ratio	Normal	30,389 (73.1%)
(categories)	Overweight	10,115 (24.3%)
	Missing	1077 (2.6%)
Systolic Blood Pressure	Mean (SD)	139 (18.8)
(mmHg)	Median [Min, Max]	138 [76.5, 238]
	Missing	6437 (15.5%)

See Methods for details on UK Biobank fields corresponding to the above traits.

### Risk factors

#### Observational associations

**Figure 1.**
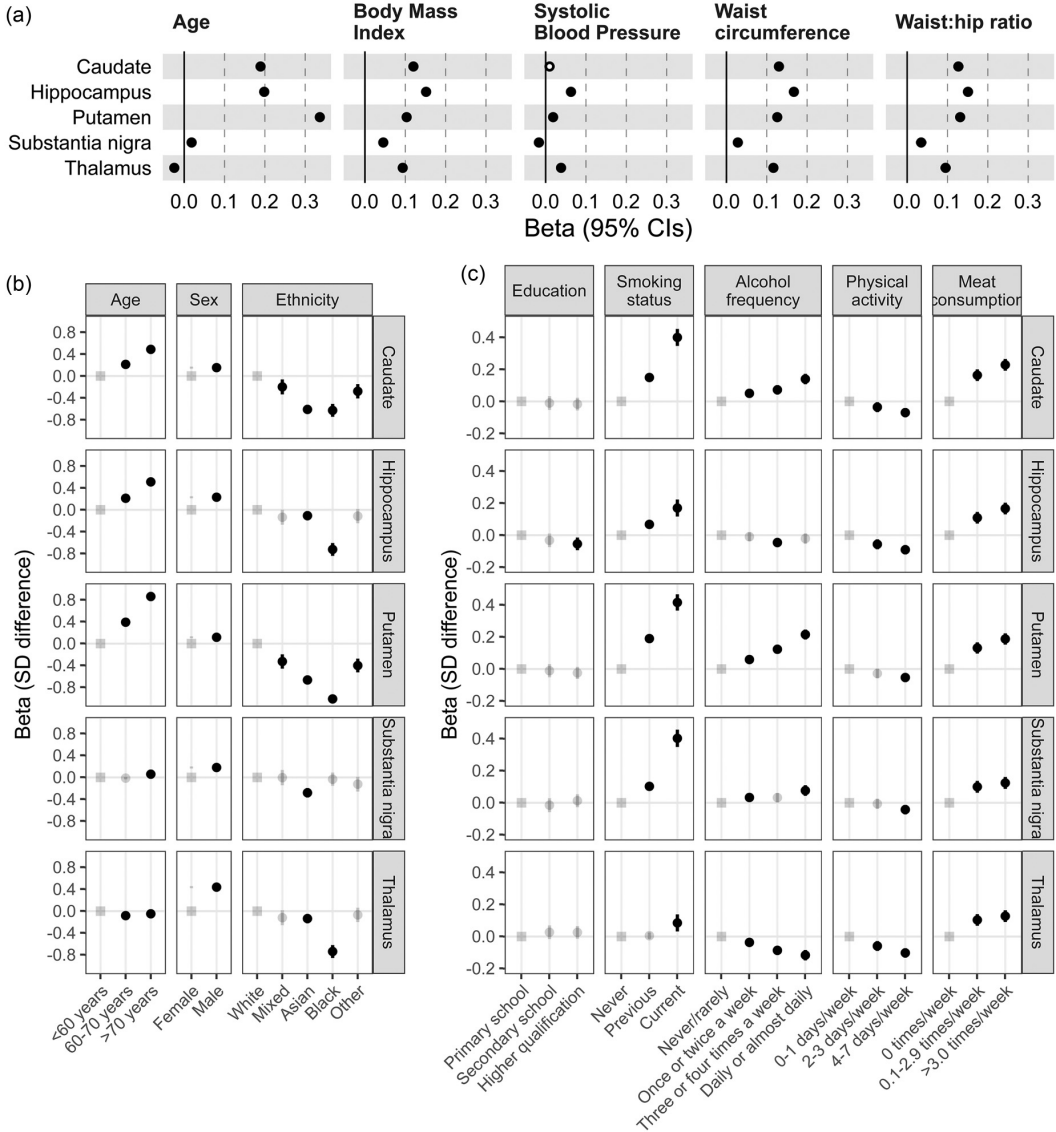
Participant characteristics at MRI assessment, associations with subcortical QSM. Results are from linear regression models in up to 41,581 UK Biobank participants, adjusted for age at MRI assessment, sex, and MRI assessment center. (a) Prevalent associations between quantitative traits and QSM values (beta = standard deviation [SD] difference in QSM per SD of the trait). (b, c) Prevalent associations between categorical traits and QSM values (beta = SD difference in QSM compared to the reference category [indicated with the ‘square’]: semi-transparent points indicate non-significant associations after multiple testing [Benjamini Hochberg p > 0.05]). Note axis scale differs between panels b and c. Lines are the 95% confidence intervals. See Supplemental Table 3 and Methods for details, including the UK Biobank fields used (some categories have been combined e.g., highest education level attained).

Age was strongly, positive associated with QSM in the putamen, hippocampus and caudate (putamen standard deviation change per year [beta] = 0·043: 95% Confidence Intervals 0·042 to 0·045, p = 2*10^−1128^), moderately in the substantia nigra (0·002: 0·001 to 0·004, p = 2*10^−4^), yet negatively in the thalamus (−0·003: −0·004 to −0·002, p = 4*10^−7^). QSM values were higher in males in all 5 regions (e.g., thalamus beta = 0·44: 0·42 to 0·46, p = 5*10^−454^). BMI, waist circumference, and waist:hip ratio were strongly, positively associated with QSM in all 5 regions (Figure 1(a); Supplemental Table 3).

Self-reported participant characteristics were also strongly associated with subcortical QSM in the 5 regions (Figure 1(b) and (c)). Self-reported ethnic background had the largest effect: black participants had >1 SD lower QSM in the putamen, compared to those self-reporting white ethnicity (beta = −1·01: −1·12 to 0·90, p = 5*10^−68^). QSM values were higher in participants self-reporting higher red/processed-meat intake, and in current smokers. Higher physical activity was associated with lower QSM. Higher alcohol intake was associated with higher iron in 3 regions, yet lower in the thalamus (beta = −0·12: −0·15 to −0·08, p = 3*10^−12^). All reported associations were significant after adjustment for multiple statistical testing (Supplemental Table 3).

**Figure 2.**
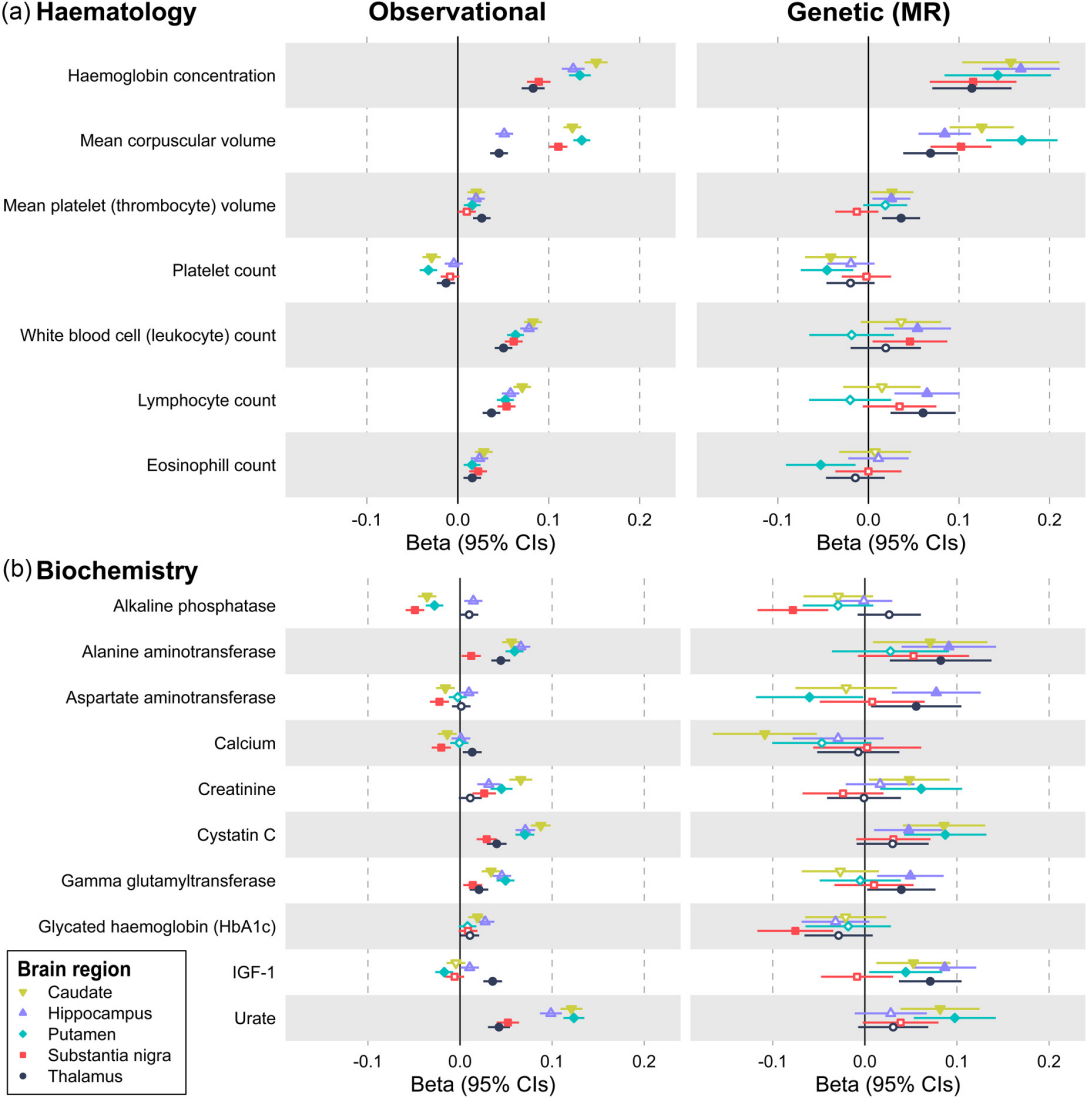
Baseline biomarkers with genetic evidence for a causal effect on QSM brain iron. Observational associations are from linear regression models in up to 39,544 UK Biobank participants, adjusted for age at baseline assessment, age at MRI assessment, sex, baseline assessment center, and MRI assessment center. The beta is the standardized coefficient, representing the standard deviation (SD) difference in QSM at MRI assessment per standard deviation increase in baseline biomarker. Genetic associations are from Mendelian randomization analysis. The Beta is the MRlap corrected IVW estimate (i.e., the beta per SD of genetically instrumented exposure, corrected for sample overlap and weak instrument bias). Only hematology and biochemistry parameters with a significant MR effect on QSM in at least one region after Benjamini-Hochberg FDR correction are shown. See Methods and Supplemental Table 4 and 7 for details.

From the baseline hematology panel (measured 8–14 years prior to MRI), the number of white blood cells (and subtypes) were associated with higher QSM-estimated iron in all 5 subcortical regions (Figure 2(a); Supplemental Table 4). Additionally, higher hemoglobin, mean red cell volume, and other parameters, yet lower red cell distribution width and platelet counts, were associated with higher QSM. From the baseline biochemistry panel, higher triglycerides, urate, and cystatin c were among those positively associated with QSM, whilst HDL cholesterol had the largest negative effect (Figure 2(b); Supplemental Table 4).

Within the 2923 proteins assayed at baseline, 107 were significantly (Bonferroni-corrected p < 1·7*10^−5^) associated with at least one QSM phenotype in the 5222 participants with MRI and proteomics data (Supplemental Figure 1; Supplemental Table 5). Brevican core protein (BCAN) had the strongest association (beta-putamen = −0·175: −0·20 to −0·15, p = 1*10^−30^). Associated proteins included those implicated in neurology (e.g., OMG), iron metabolism (TF), metabolism (LEP), and inflammation (TNFRSF10B).

#### Genetic associations

Significant observational associations were repeated using MR, where possible, to estimate causal effects.

Higher BMI was casually associated with higher brain QSM in all brain areas (Table 2; Supplemental Table 6). Waist circumference and waist:hip ratio were associated with higher QSM in the putamen, hippocampus, caudate and thalamus, but not the substantia nigra. Results do not support causal effects (FDRp > 0.05) for SBP, HDL, LDL or triglycerides on QSM in these regions (Supplemental Tables 6 and 7). Results support causal effects for hematology and biochemistry markers (FDRp < 0.05), e.g., hemoglobin and mean corpuscular volume with all regions (Figure 2; Supplemental Table 7). IGF1 was associated with caudate, hippocampus and thalamus QSM, alanine aminotransferase and lymphocyte count with hippocampus and thalamus QSM and cystatin C, platelet count and urate with caudate and putamen QSM (Supplemental Table 7). Other markers had region-specific associations: aspartate, glutamyl transferase, monocytes, neutrophils and white cell count were associated with hippocampus QSM; mean platelet thrombocyte volume was only associated with thalamus QSM; alkaline phosphatase levels and hba1c were only associated with substantia nigra QSM; creatinine with putamen QSM; and calcium with caudate QSM (Figure 2 and Supplemental Table 7).

**Table 2.** Causal effects on QSM brain iron estimated by Mendelian randomization.

Exposure	Outcome region	Beta	SE	p
BMI	Caudate	0.165	0.027	4.98E-10
	Hippocampus	0.158	0.025	2.99E-10
	Putamen	0.145	0.027	6.15E-08
	Substantia nigra	0.079	0.028	4.84E-03
	Thalamus	0.117	0.027	1.45E-05
Waist	Caudate	0.177	0.030	2.38E-09
circumference	Hippocampus	0.207	0.026	2.76E-15
	Putamen	0.212	0.031	1.45E-11
	Substantia nigra	0.040	0.033	2.28E-01
	Thalamus	0.133	0.030	7.04E-06
Depression	Caudate	0.364	0.117	1.86E-03
	Hippocampus	0.161	0.130	2.16E-01
	Putamen	0.258	0.132	5.15E-02
	Substantia nigra	0.460	0.129	3.71E-04
	Thalamus	−0.070	0.114	5.41E-01
Type 2 diabetes	Caudate	0.116	0.030	9.55E-05
	Hippocampus	0.108	0.025	1.66E-05
	Putamen	0.093	0.029	1.42E-03
	Substantia nigra	0.043	0.031	1.58E-01
	Thalamus	0.060	0.025	1.84E-02
Iron deficiency	Caudate	−0.358	0.245	1.43E-01
anemia	Hippocampus	−0.410	0.244	9.36E-02
	Putamen	−0.164	0.332	6.21E-01
	Substantia nigra	−0.802	0.235	6.63E-04
	Thalamus	−0.278	0.239	2.44E-01
Osteoarthritis	Caudate	0.292	0.098	2.76E-03
	Hippocampus	0.168	0.101	9.62E-02
	Putamen	0.076	0.097	4.34E-01
	Substantia nigra	0.108	0.109	3.21E-01
	Thalamus	0.277	0.112	1.30E-02
Osteoporosis	Caudate	−0.111	0.048	2.12E-02
	Hippocampus	0.001	0.045	9.83E-01
	Putamen	−0.067	0.063	2.87E-01
	Substantia nigra	−0.257	0.047	3.74E-08
	Thalamus	0.045	0.049	3.53E-01
Psoriasis	Caudate	0.064	0.014	5.36E-06
	Hippocampus	−0.004	0.017	7.98E-01
	Putamen	0.056	0.017	9.03E-04
	Substantia nigra	0.025	0.015	1.04E-01
	Thalamus	−0.045	0.018	1.37E-02
Transient ischemic	Caudate	0.599	0.239	1.24E-02
attack	Hippocampus	0.541	0.231	1.95E-02
	Putamen	1.137	0.286	7.06E-05
	Substantia nigra	0.378	0.238	1.13E-01
	Thalamus	0.120	0.238	6.14E-01

Results from MRlap. The Beta, Standard Error (SE), and p-value are the estimates corrected for sample overlap and weak instrument bias. All exposures listed were significantly associated with QSM in at least one region after adjustment for multiple statistical testing (Benjamini-Hochberg p < 0.05). See Results and Supplemental Tables 6 and 12 for further details.

For behavioral phenotypes where a GWAS of the trait was unavailable, we used the closest phenotype where a GWAS was available (see Methods). Higher educational attainment was associated with lower caudate, hippocampus and putamen QSM. “Ever smoked” and higher alcohol consumption were causally associated with higher thalamus QSM, whereas overall physical activity was not associated (Supplemental Table 6).

Of 2018 protein assays with a reported cis-variant^17^ 110 were significantly associated with at least one QSM trait from published GWAS^3^ after FDR correction (Benjamini-Hochberg-adjusted p < 0·05) (Supplemental Table 8). Two proteins were associated with QSM in all five brain regions (CDSN and DBI), 2 proteins with 4 regions, 15 with 3 regions, 22 with 2 regions, and 69 with one region only. Conversely, the QSM-region with most significant protein associations was the putamen (n = 52), followed by the substantia nigra (n = 46), caudate (n = 43), thalamus (n = 23) and hippocampus (n = 12, Supplemental Table 8).

Using FUMA, we identified the following tissues where the above proteins with a causal effect on QSM (MRI-estimated brain iron) are differentially expressed (FDRp < 0·05): adipose tissue, spleen, lung, testis and esophagus (Supplemental Table 9). Additionally, gene ontology biological processes over-represented included iron metabolism, immune process and its regulation, inflammation and cytokine production, cell adhesion, and regulation of gene expression (Supplemental Table 10).

### Long-term conditions

#### Observational associations

**Figure 3.**
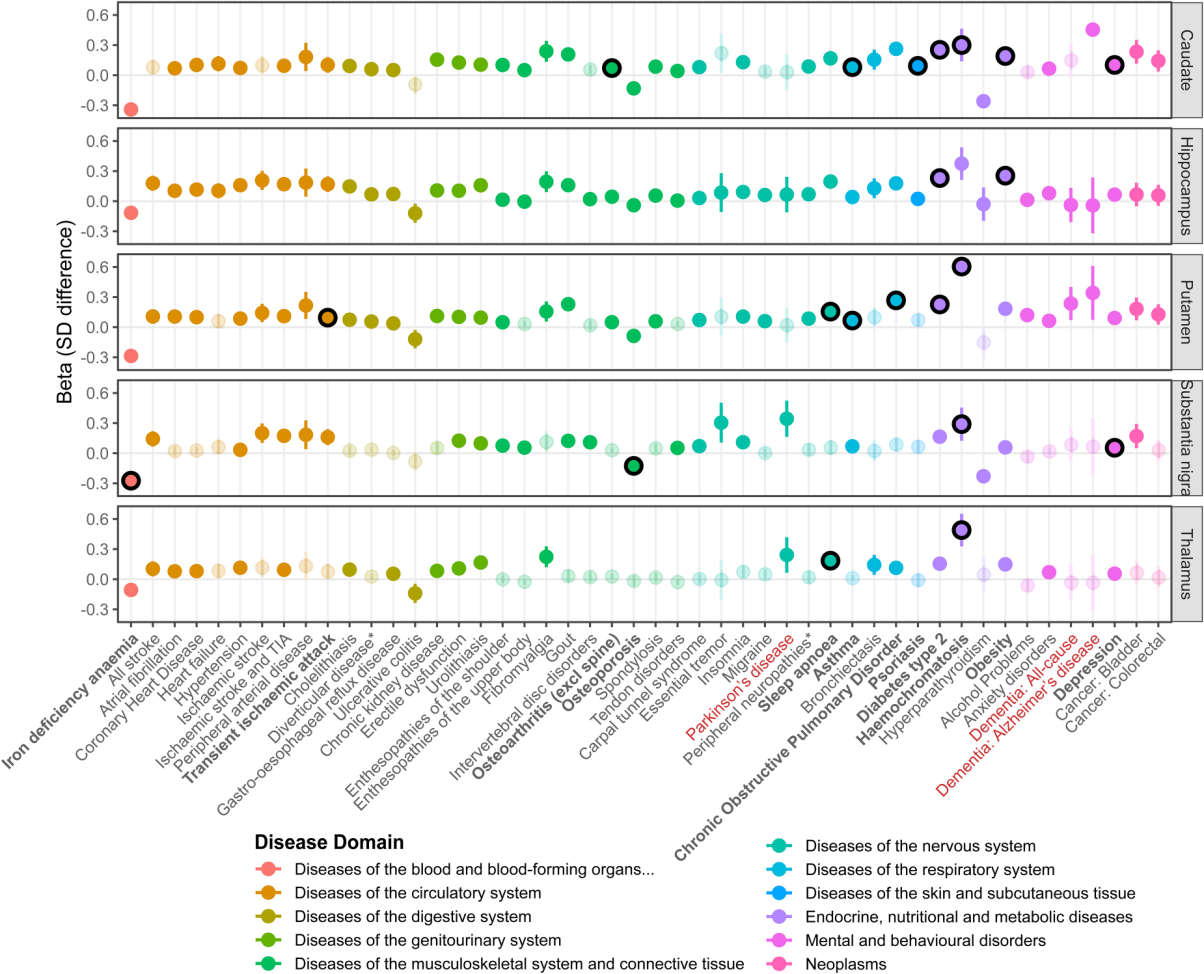
Prevalent diseases associated with subcortical QSM. Results from linear regression models in 41,581 UK Biobank participants, adjusted for age at MRI assessment, sex, and MRI assessment center. The beta represents the standard deviation (SD) difference in QSM between the diagnosed participants and those without a diagnosis at the time of MRI assessment. Diseases are colored according to their primary ICD-10 chapter domain. Semi-transparent points indicate the association was non-significant after adjustment for multiple testing (Benjamini-Hochberg-adjusted p > 0.05). Lines are the 95% Confidence Intervals. Black outlines (and bold disease names) indicate a significant causal effect was identified in Mendelian randomization analysis (FDR p < 0.05). 48 of the 88 diseases tested are shown, where there was a significant association with at least one QSM region: for further details (including full disease definitions where an abbreviated form was shown in the plot *) and results see Supplemental Table 11.

We estimated associations between 88 prevalent long-term conditions (diagnoses) and QSM in the 5 subcortical regions. 48 were associated with at least one region (Figure 3 and Supplemental Table 11) after multiple testing correction (FDRp < 0·05): conditions where QSM was higher in diagnosed individuals included AD, PD, type-2 diabetes, and hemochromatosis. Four conditions were associated with lower QSM in at least one region: anemia, osteoporosis, ulcerative colitis, and hyperparathyroidism.

Most associations remained consistent after adjustment for additional covariates (ethnic background, education [highest qualification], smoking status, alcohol [days per week], physical activity [days per week with moderate activity], meat consumption [days per week with red or processed meat intake], waist circumference, systolic blood pressure). Using Fisher's Z to formally test for significant differences in estimates between Model 1 and Model 2, associations between diagnosed obesity and QSM were most attenuated (expected, given the adjustment for waist circumference), whilst hypertension, type-2 diabetes, gout, COPD and depression were only partially attenuated (Supplemental Figure 2; Supplemental Table 11).

We also investigated whether prevalent disease associations differed between males and females (using Fisher's Z): the majority of disease-QSM associations are not significantly different (Supplemental Figure 3; Supplemental Table 11), yet some associations were stronger in females compared to males (including tendon disorders, enthesopathies, bursitis, migraine, colorectal cancer, anxiety disorders, and depression), where others were stronger in males (including schizophrenia, type-2 diabetes, hypertension, and non-melanoma skin cancer).

#### Genetic associations

MR of the disease-QSM associations support causal effects of genetic liability to type-2 diabetes with caudate, hippocampus and putamen QSM, and psoriasis on caudate and putamen QSM (Figure 3; Table 2; Supplemental Table 12). Results also support causal effects of: depression on caudate and substantia nigra QSM; asthma, COPD, sleep apnea, and TIA on putamen QSM; osteoarthritis and intervertebral disk on caudate QSM; osteoporosis, sleep apnea, and anemia on substantia nigra QSM; and migraine on thalamus QSM.

For hemochromatosis we investigated the known disease-causing variants *HFE* C282Y and H63D, and estimated associations with QSM, comparing levels in the YY homozygotes (n = 217) to the CC/HH individuals (i.e., without either allele, n = 22,961). The YY homozygotes had higher QSM in all 5 regions, with the largest effect in the putamen (beta = 0·90: 0·77 to 1·02, p = 2*10^−45^). Results were consistent across males and females, and in sensitivity analyses excluding those with diagnosed hemochromatosis (Supplemental Table 13).

### Mendelian randomization sensitivity analyses

Heterogeneity in variant-effects is common, as indicated by statistically significant Q-statistics, suggesting pleiotropic pathways (Supplemental Table 14). For example, some baseline hematology and biochemistry markers such as for hemoglobin and mean corpuscular volume have MR-Egger intercept p < 0.05. Other results warranting cautious interpretation and further investigation include alkaline phosphatase, HbA1c, and IGF1 (Supplemental Table 15). Extended plots and results available https://github.com/AGEexeter/paper-brain_iron_causes.

We investigated the effect of outliers using leave-one-out analysis. For calcium there is a single larger-effect variant rs34408666 (Supplemental Figure 4) yet the estimate was consistent when excluding rs34408666 (caudate-beta = −0·102, se = 0·029, p = 0·0005). Similarly, removing larger-effect variants for alkaline-phosphatase∼substantia-nigra (rs10916988), urate-caudate and putamen (rs4697708), asthma-caudate and putamen (rs1047989), COPD-putamen (rs11852372), diabetes-caudate and putamen (rs72826075), did not meaningfully alter results (see Supplemental Figures 4–12). For other baseline hematology and biochemistry results there was no evidence of pleiotropy (Supplemental Table 15).

For long term conditions and risk factors there was no evidence that pleiotropy affected out results except for WHR on substantia nigra, depression and caudate, and osteoporosis on substantia nigra, where the MR-Egger intercept was significant (Supplemental Table 16). For the risk factors we only found evidence of pleiotropy for WHR-thalamus (Supplemental Table 17).

## Discussion

We here demonstrate that adiposity (both BMI and waist circumference), causally increases MRI-estimated brain iron in subcortical regions implicated in dementia or PD. We show that the pathway from adiposity to brain iron is linked to inflammation and suggest a role for impaired glycemic control. Our findings do not support causal effects for other cardiovascular risk factors such as high blood pressure.

Adiposity is observationally associated with brain iron in aging mice.^11^ Visceral adiposity increased inflammation, hepcidin production, and brain iron deposition,^24^ highlighting complex interactions between hepcidin in the brain and iron, though evidence in humans is needed. Our genetic evidence supports a causal effect of adiposity (using BMI and waist circumference measures) on subcortical iron deposition. Though we lack data on hepcidin, this mechanism is supported by our report of the role of central adiposity in hemochromatosis,^25^ where HFE (homeostatic iron regulator) is responsible for detecting serum iron and regulates hepcidin.

The role of inflammation on hepcidin and iron is complex: inflammation increases hepcidin production leading to intracellular iron retention, which can cause anemia,^2^ yet inflammation activates microglia in the brain, leading to iron deposition.^1^ A role for inflammation in brain iron deposition is supported by our proteomics results where genetically predicted higher IL6R (expressed on leukocytes and regulates the cellular response to the multifunctional cytokine IL6^26^) was associated with higher MRI-estimated iron in the caudate. Pathway analysis also implicated the immune system and its regulation in brain iron deposition, as well as our finding that genetic liability to psoriasis and asthma increased QSM-estimated brain iron.

Consistent with previous literature linking brain iron deposition and neurodegenerative disease^1^ we observed that participants diagnosed with AD or PD had higher MRI-estimated brain iron, despite small numbers with diagnoses (n = 47 and n = 117, respectively). Interestingly, different regions are implicated, highlighting specific disease pathology. We did not find evidence supporting a causal effect of liability to dementia or PD on QSM, consistent with the disease being secondary to iron deposition. We previously reported that brain iron in 4 of the subcortical regions studied here have a causal effect on either PD (caudate, putamen, and substantia nigra) or non-Alzheimer's dementia (thalamus and putamen).^3^ Though we did not observe higher iron in the participants diagnosed with non-Alzheimer's dementia (or vascular dementia specifically), the number are very low (n = 77 and n = 22, respectively) resulting in wide confidence intervals, rendering it difficult to draw firm conclusions regarding non-significant associations. Overall, our results are consistent with subcortical iron deposition occurring in participants diagnosed with dementia or PD and support a causal role of iron deposition. This is consistent with some older clinical trials, for example a study of the iron chelator deferoxamine reported that therapy may slow progression of dementia,^27^ but in contrast to a recent trial of the iron chelator deferiprone which accelerated cognitive decline in symptomatic patients.^28^ This is in the context of a recent (2024) review of iron chelators and AD clinical trials, concluding there were no significant positive outcomes reported.^5^ Clearly, more work is needed to establish the complex causal relationships of iron metabolism in dementia. Our cohort analysis differs from the trials, as we predominantly study individuals without dementia symptoms or cognitive impairment, and may explain some differences observed: earlier interventions to prevent iron overload may be more effective than intervening to reduce iron in symptomatic individuals, after irreversible damage may have already occurred. Additionally, our analysis does not rule out other important roles for iron in dementia pathology, indeed prior studies have highlighted iron-responsive-like elements (IREs) in neurodegenerative ferroptosis.^29^ The MRI-estimated QSM phenotype available is not able to capture IREs.

The role of iron overload in dementia onset is inconsistent, with studies reporting that carriers of *HFE* genetic variants implicated in the iron-overload disease hemochromatosis have increased dementia risk: yet the results are mixed, with some reports of the *HFE* p.H63D with AD^30^ and others of *HFE* p.C282Y (the main iron-increasing mutation) with non-AD dementias^31^ In our previous papers we used genetic analysis methods (Mendelian randomization) which supported causal effects of serum iron (transferrin saturation)^4^ and MRI-estimated brain iron (thalamus)^3^ on liability to non-AD dementia but not AD. Overall, our results are consistent with the literature that genetic variants linked to iron overload increase dementia risk,^32^ though further work is needed to understand the roles in AD and non-AD pathologies.

Previous observational studies report associations between glycemia and brain iron.^8–10^ Our results support a causal effect of genetic liability to type-2 diabetes and iron deposition in the caudate and putamen. We speculate that microvascular dysfunction may be the mechanism, as opposed to macrovascular disease, because we found no associations between coronary heart disease or systolic blood pressure with brain iron. Previous reports that impaired glucose metabolism and energy supply due to insulin resistance can result in neurodegeneration (brain glucose hypometabolism), especially in regions with high metabolic demands such as subcortical regions studied.^33^

Our observational results show that patients diagnosed with depression have higher MRI-estimated brain iron, supporting previous studies implicating iron homeostasis in depression.^34^ We extend this by showing that genetic liability for depression is associated with higher brain iron, supporting a causal association. Given our above findings of a causal link between higher BMI and brain iron, and published evidence of a genetic link between BMI and depression,^35^ the relationship between depression, adiposity, and iron, warrants further investigation.

As expected, we found strong evidence that iron-related metabolism has an important role in determining MRI-estimated brain iron (QSM), with both observational and genetic evidence. We previously reported that the hemochromatosis-associated *HFE* C282Y variant increased MRI-estimated brain iron estimated by the older R2* method,^7^ which we here confirm with the newer QSM method.^6^ Our results highlight that a balance between high and low iron has to be achieved: we show that genetic liability for anemia is associated with lower brain QSM, and whilst it is known that high iron is a risk factor for neurodegenerative diseases^3,4^ it has also been shown that pathologically low iron is a risk factor,^36^ supported by studies linking depression and anemia.^37^ Though it is important to note there may be discrepancies between studies of serum iron and brain iron, where in the former we expect more profound U-shape relationships with adverse outcomes (anemia versus iron overload), and in the latter there is generally low iron across the brain which accumulates with age, though regional heterogeneity exists.^36^ The association between red meat (a high-iron food) consumption and brain QSM has been previously reported,^6^ which we extend by demonstrating the independent effects of red/processed meat consumption on brain iron deposition after adjustment for many other health and socio-economic factors.

Osteoporosis and hyperparathyroidism diagnoses were also associated with lower MRI-estimated iron in the substantia nigra and caudate. Additionally, we observe that higher baseline calcium was associated with lower iron in the same regions. Calcium, like iron, has essential functions but high levels can be harmful.^38^ The relationship between calcium and iron is complex: the parathyroid glands secrete parathyroid hormone (PTH) to regulate calcium levels in the blood, and iron deposition in the glands can cause PTH dysregulation.^39^ Additionally, higher dietary calcium can inhibit non-heme iron absorption via shared transporters (e.g., DMT1).^40^ A study of 106 AD patients found higher iron in the cerebral spinal fluid yet lower calcium, possibly due to calcium reuptake by cells in response to the higher iron, leading to cell death.^41^ Our genetic analysis supports a causal effect for osteoporosis and serum calcium on lower brain iron. Statistical power for the analysis of hyperparathyroidism was limited. Our results highlight the important dynamic between iron and calcium, and the need for further work to understand the links to iron deposition in the substantia nigra and caudate.

We used the QSM method for quantifying brain tissue magnetic susceptibility because it has several advantages over older T2* methods, especially differentiating between paramagnetic and diamagnetic substances.^6^ Brain iron is stored primarily as Fe3+ bound to ferritin but can also be found in amyloid plaques.^42,43^ Amyloid-β promotes the reduction of Fe3+ to magnetite, which has greater magnetic susceptibility.^43,44^ We are not able to differentiate ferritin-stored iron or amyloid magnetite. Nonetheless, QSM quantifies the mean intravoxel susceptibility, indicating the amount of susceptibility sources, such as iron.

UKB is an exceptional resource but has limitations. Principally, the 5% response rate at baseline, the lack of ethnic diversity (>95% of participants self-reported their ethnic background as White), and known higher socio-economic demographic than the general population; yet pathological findings are generalizable to the UK population.^45^ QSM estimated brain iron from MRIs may be influenced by other factors.^6^ Diagnoses are primarily from secondary care (hospital inpatient admissions); future work integrating more primary care diagnoses are required, especially for non-hospitalizing conditions. MR evidence can support causal effects of risk factors or diseases on brain iron, but relies on strong assumptions and can be biased due to pleiotropy: we investigated this in sensitivity analysis and overall the genetic and observational findings are consistent, but results should be interpreted cautiously, especially where there is evidence of heterogeneity.

To conclude, we have shown that higher adiposity and inflammation, and lower calcium, are significant causal risk factors for MRI-estimated iron deposition in specific subcortical brain regions. Genetic liability to type-2 diabetes, depression, and other conditions, also affect subcortical iron deposition. The role of adiposity reducing interventions on brain iron should be investigated. The relationship between brain iron, osteoporosis, calcium, and hyperparathyroidism warrants further investigation. Generalizability of results to more diverse populations is also warranted.

## Supplemental Material

sj-docx-1-alz-10.1177_13872877251375432 - Supplemental material for Predictors of MRI-estimated brain iron deposition in dementia and Parkinson's disease-associated subcortical regions: Genetic and observational analysis in UK Biobank Supplemental material, sj-docx-1-alz-10.1177_13872877251375432 for Predictors of MRI-estimated brain iron deposition in dementia and Parkinson's disease-associated subcortical regions: Genetic and observational analysis in UK Biobank by Francesco Casanova, Qu Tian, Daniel S Williamson, Mitchell R Lucas, David Zweibaum, Jun Ding, Janice L Atkins, David Melzer, Luigi Ferrucci and Luke C Pilling in Journal of Alzheimer's Disease

sj-xlsx-2-alz-10.1177_13872877251375432 - Supplemental material for Predictors of MRI-estimated brain iron deposition in dementia and Parkinson's disease-associated subcortical regions: Genetic and observational analysis in UK Biobank Supplemental material, sj-xlsx-2-alz-10.1177_13872877251375432 for Predictors of MRI-estimated brain iron deposition in dementia and Parkinson's disease-associated subcortical regions: Genetic and observational analysis in UK Biobank by Francesco Casanova, Qu Tian, Daniel S Williamson, Mitchell R Lucas, David Zweibaum, Jun Ding, Janice L Atkins, David Melzer, Luigi Ferrucci and Luke C Pilling in Journal of Alzheimer's Disease
