# Supplementary material for: Predictors of MRI-estimated brain iron deposition in dementia and Parkinson's disease-associated subcortical regions: Genetic and observational analysis in UK Biobank
Source: J Alzheimers Dis. 2025 Sep 15;108(1):107–18. doi: 10.1177/13872877251375432 (PMC12541117; doi:10.1177/13872877251375432)
Supplement: sj-docx-1-alz-10.1177_13872877251375432 - Supplemental material for Predictors of MRI-estimated brain iron deposition in dementia and Parkinson's disease-associated subcortical regions: Genetic and observational analysis in UK Biobank [file sj-docx-1-alz-10.1177_13872877251375432.docx]

**Supplemental Material**

**Predictors of MRI-estimated brain iron deposition in dementia and Parkinson’s disease-associated subcortical regions: Genetic and observational analysis in UK Biobank**

Specific supplementary figures mentioned in the manuscript are included here. Other supplementary results files and figures are available on our GitHub https://github.com/AGEexeter/paper-brain_iron_causes.

##
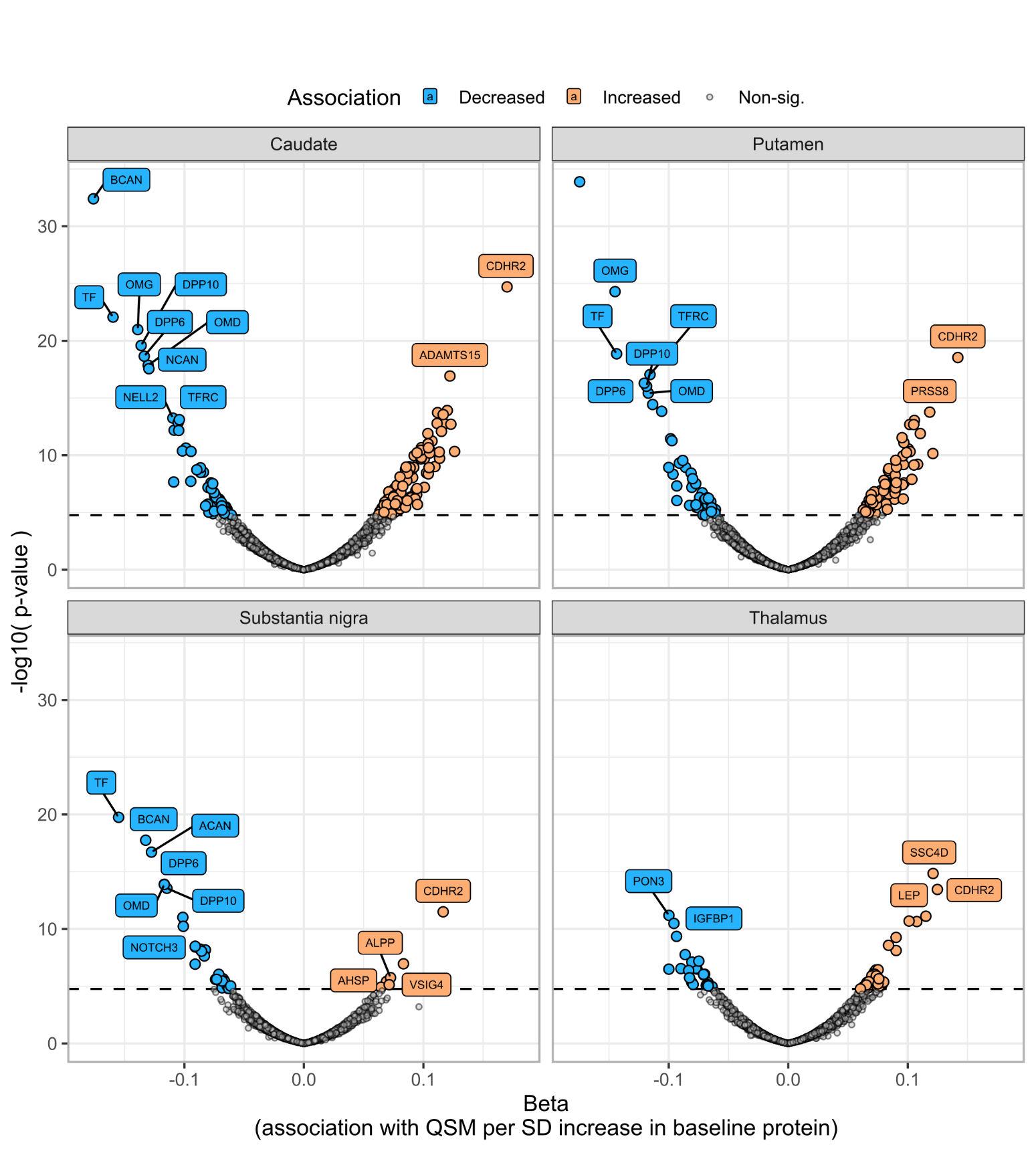
Supplemental Figure 1. Baseline proteomic associations with subcortical QSM

Results from linear regression models in up to 3,614 UK Biobank participants, adjusted for age at baseline assessment, age at MRI assessment, sex, baseline assessment center, MRI assessment center, and whether the participant was UKB-PPP consortium selected. The beta is the standardized coefficient, representing the standard deviation (SD) difference in QSM at MRI assessment per standard deviation increase in baseline proteomic measure. The horizontal line indicates significant after Bonferroni correction for multiple statistical testing. See Supplemental Table 5 for details.

## Supplemental Figure 2. Prevalent disease associations with additional adjustments


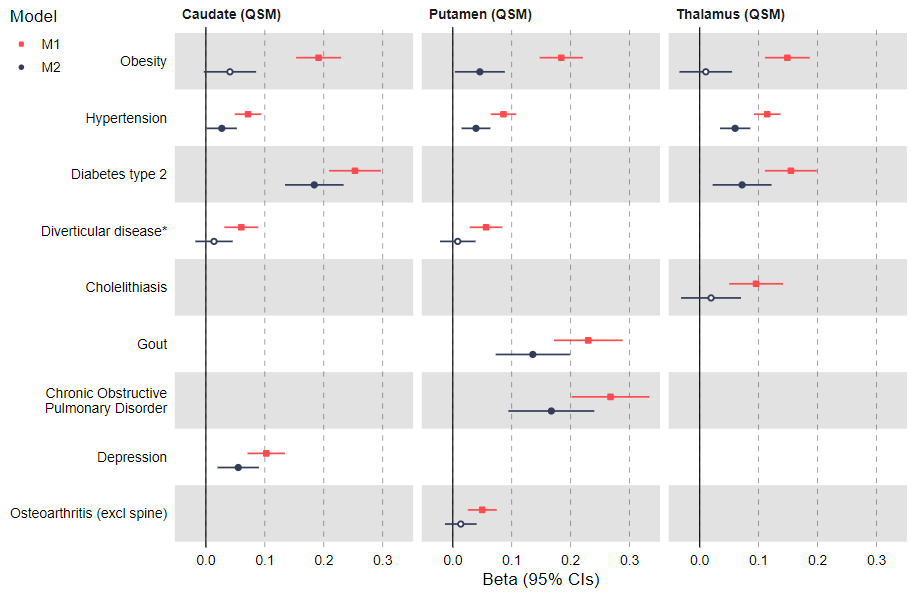


Model 1 = age, sex, assessment center

Model 2 = M1 + ethnic background + education (highest qualification) + smoking status + alcohol (days per week) + physical activity (days per week with moderate activity) + meat consumption (days per week with red or processed meat intake) + waist circumference + systolic blood pressure

Results are only shown for disease-QSM pairs where there was a significant difference between the estimates from M1 and M2 (Fisher’s Z).

##
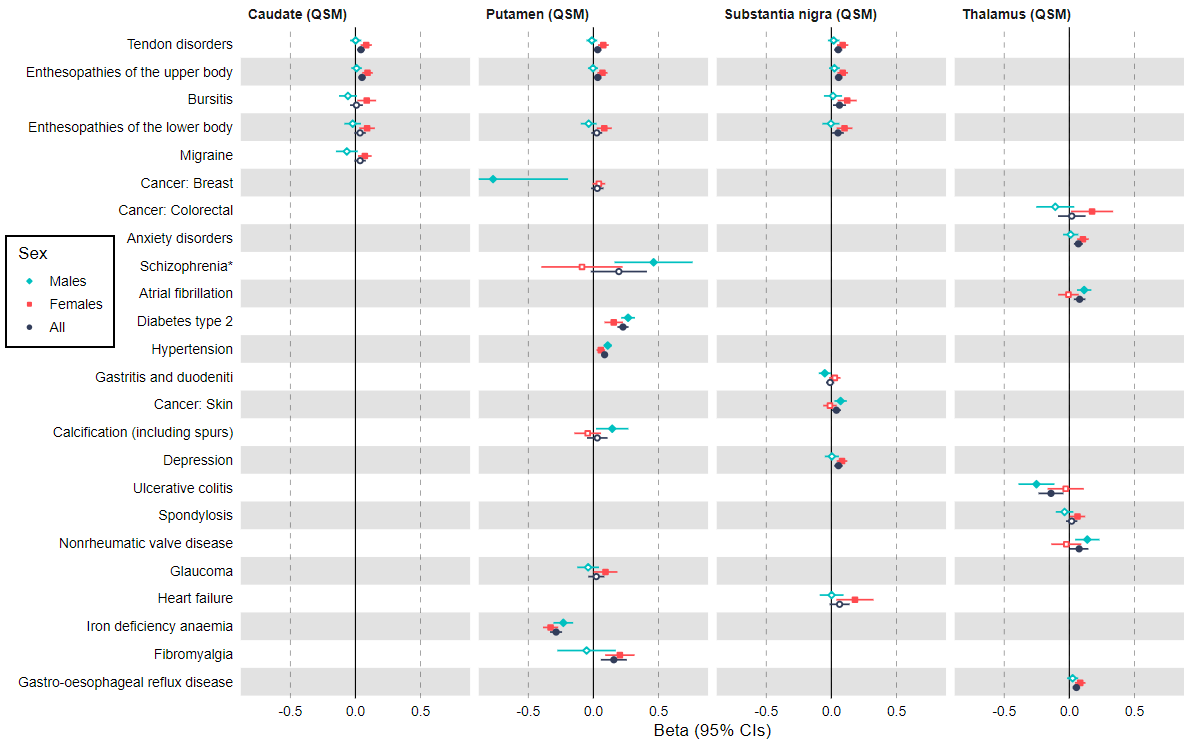
Supplemental Figure 3. Prevalent disease associations stratified by sex

Results are only shown for disease-QSM pairs where there was a significant difference between the estimates in models stratified by sex (Fisher’s Z).

## Supplemental Figure 4. Scatter plot of SNP effect on calcium and SNP effect on caudate QSM


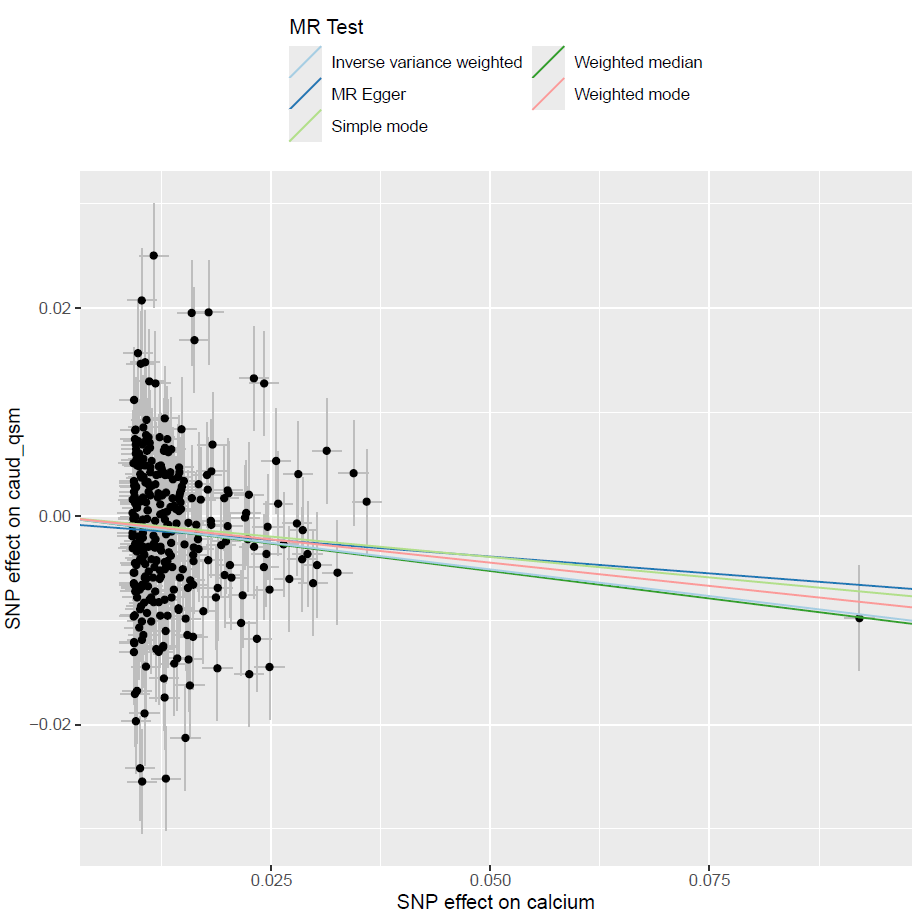


## Supplemental Figure 5. Scatter plot of SNP effect on alkaline phosphatase and SNP effect on substantia nigra QSM


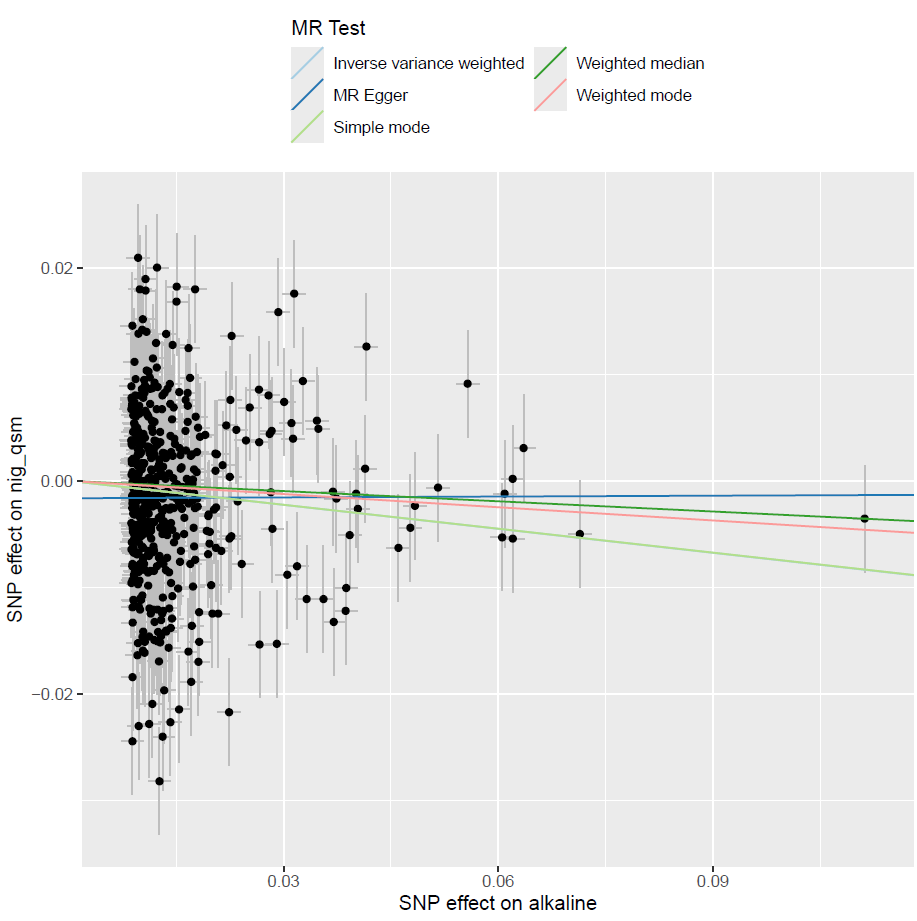


## Supplemental Figure 6. Scatter plot of SNP effect on urate and SNP effect on caudate QSM


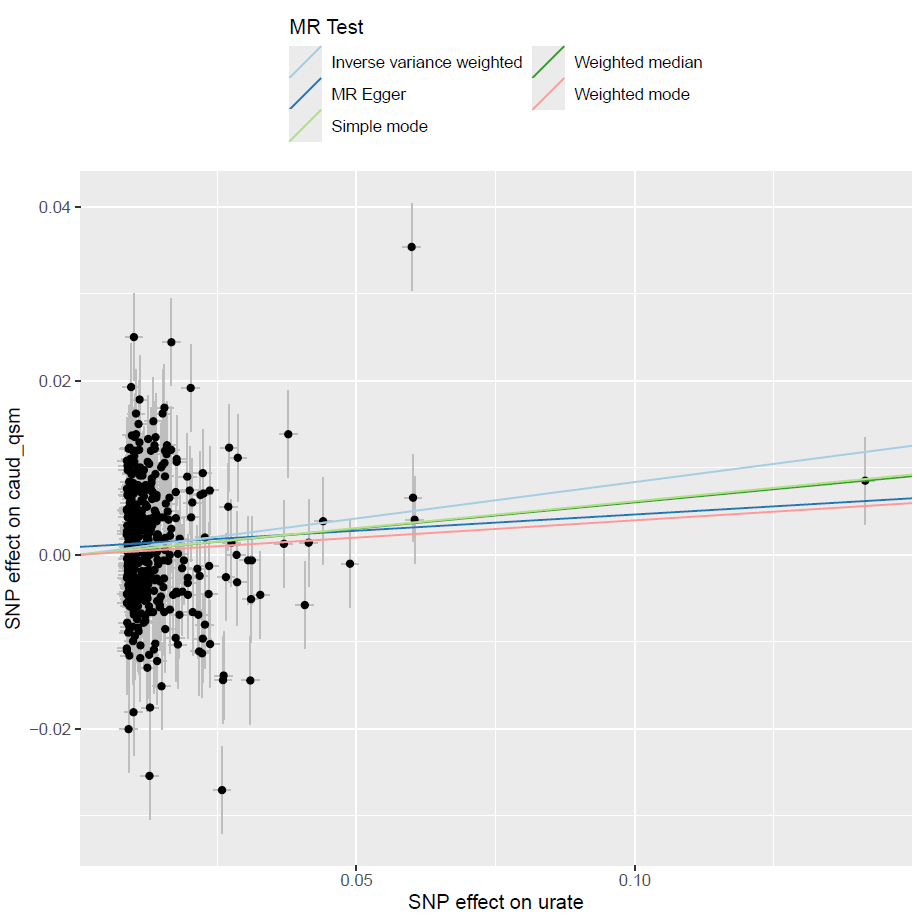


## Supplemental Figure 7. Scatter plot of SNP effect on urate and SNP effect on putamen QSM

**
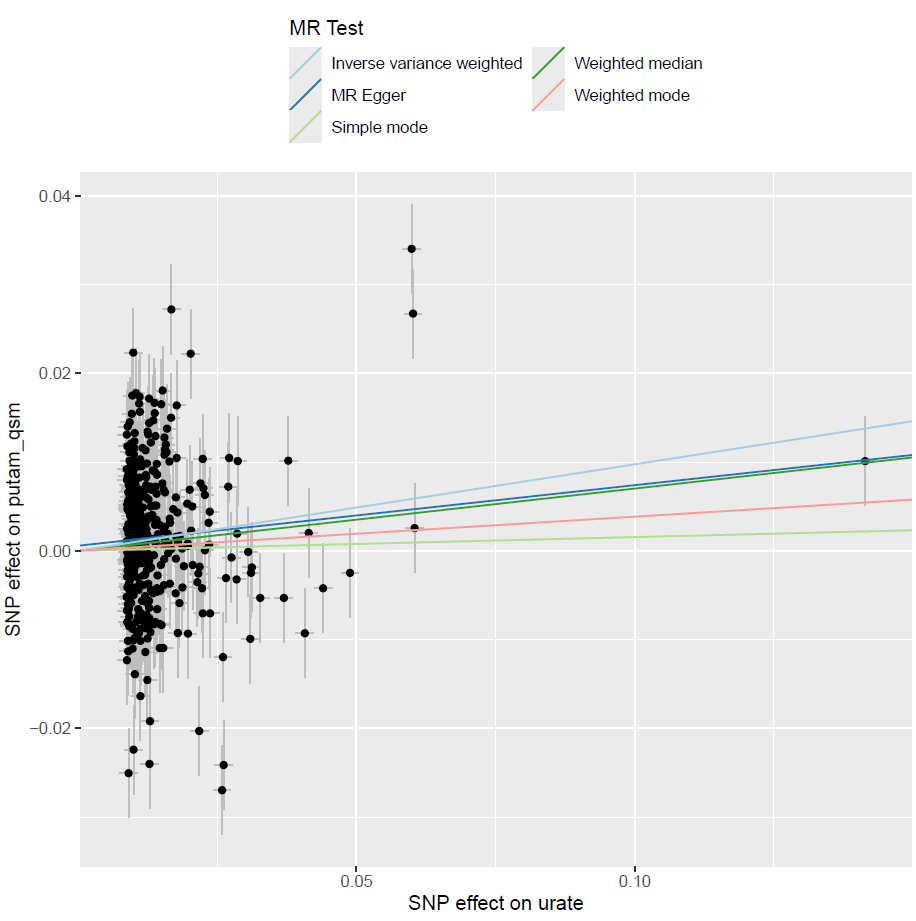
**

## Supplemental Figure 8. Scatter plot of SNP effect on asthma and SNP effect on caudate QSM

**
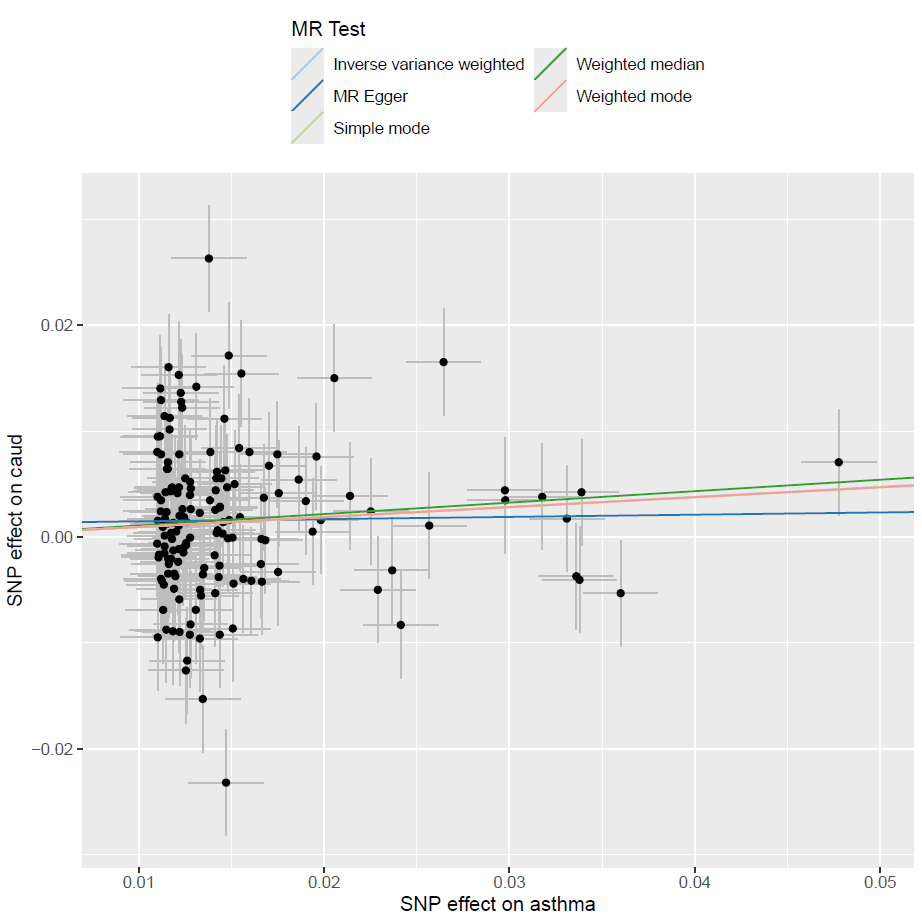
**

## Supplemental Figure 9. Scatter plot of SNP effect on asthma and SNP effect on putamen QSM

**
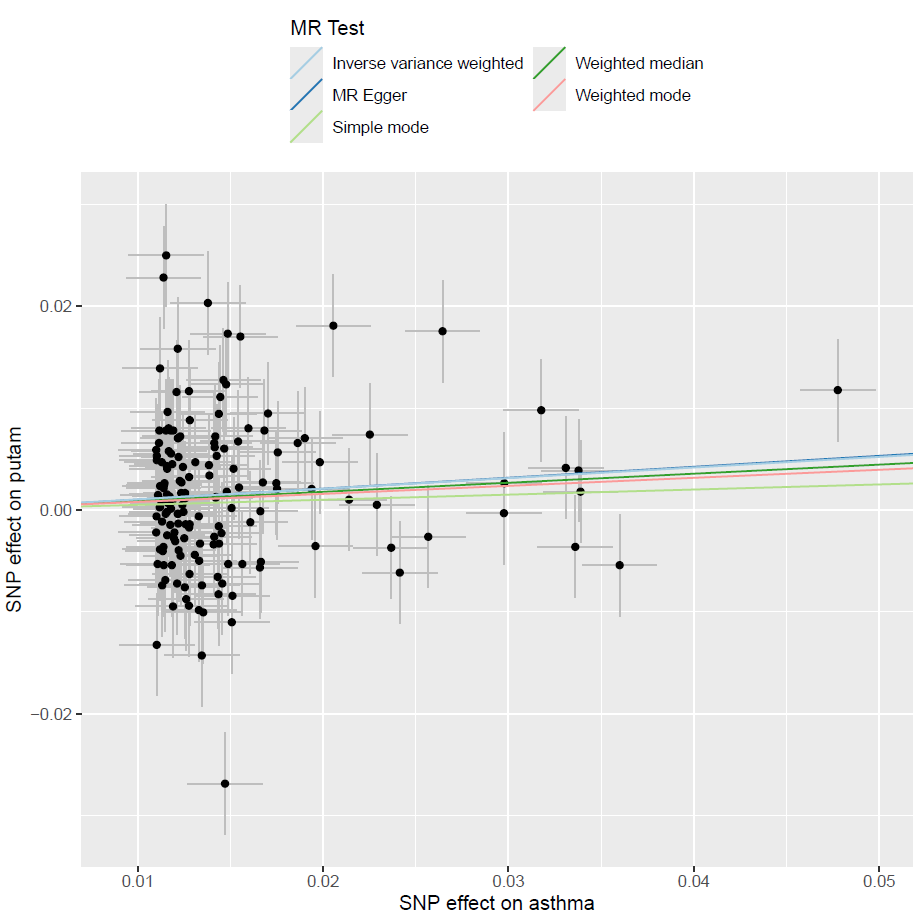
**

## Supplemental Figure 10. Scatter plot of SNP effect on COPD and SNP effect on putamen QSM

**
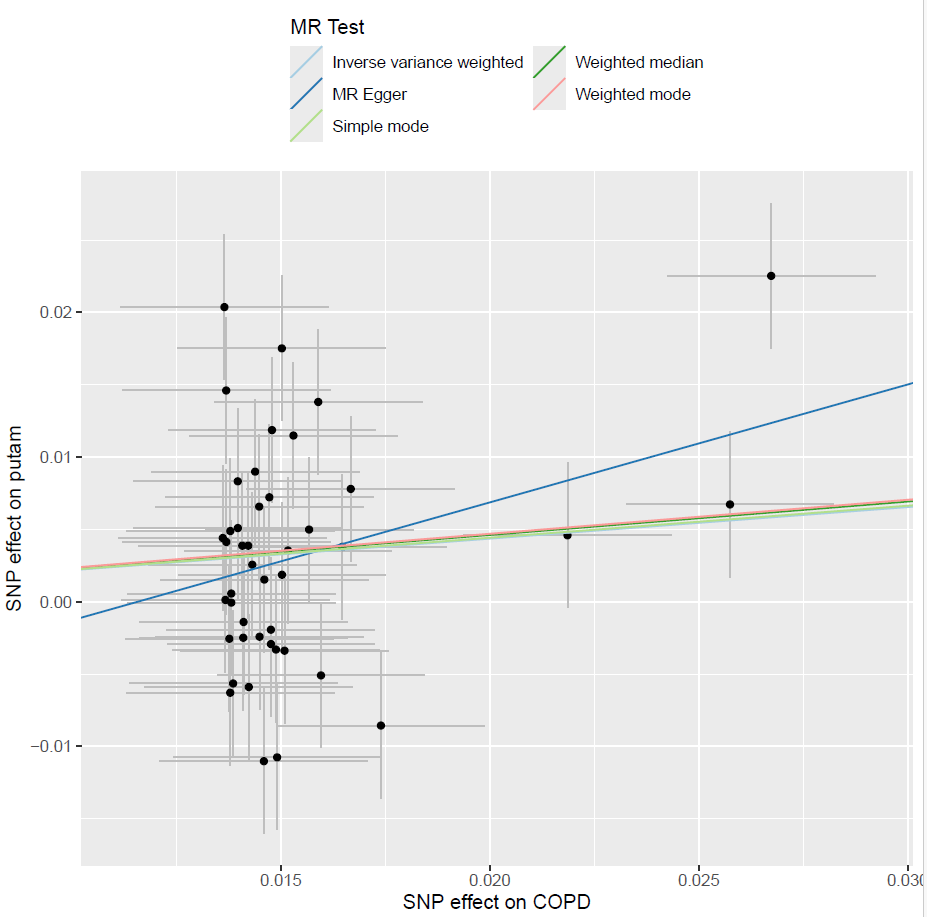
**

## Supplemental Figure 11. Scatter plot of SNP effect on type 2 diabetes and SNP effect on caudate QSM

**
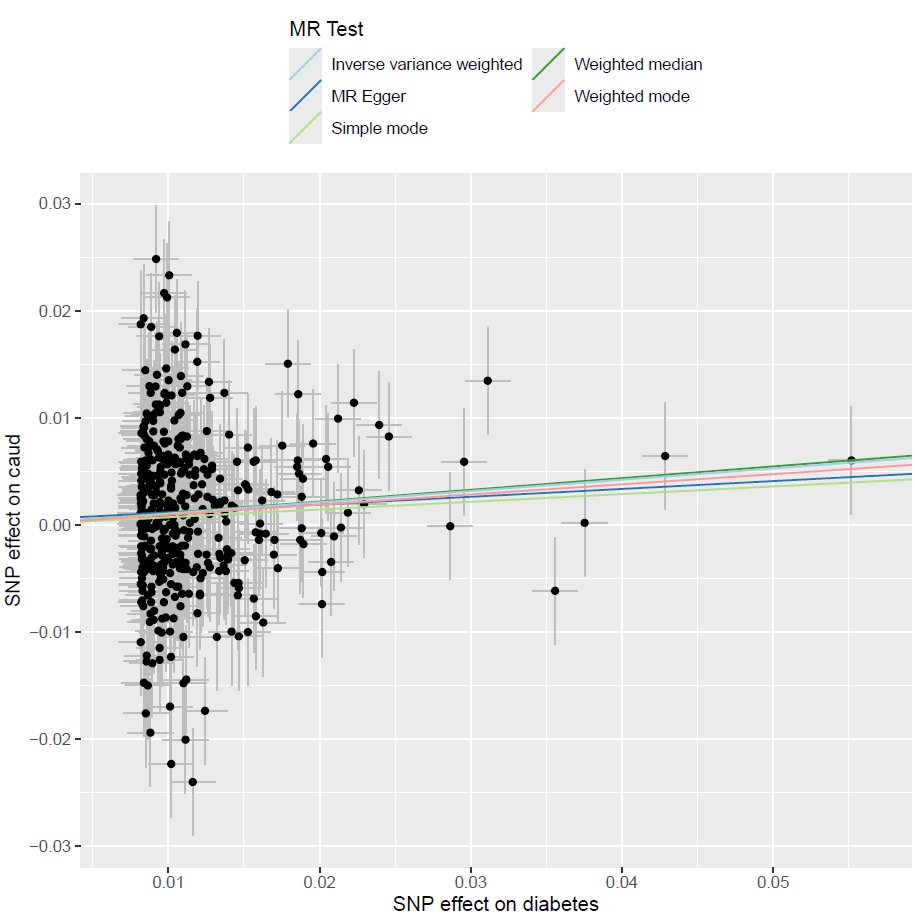
**

## Supplemental Figure 12. Scatter plot of SNP effect on type 2 diabetes and SNP effect on putamen QSM

**
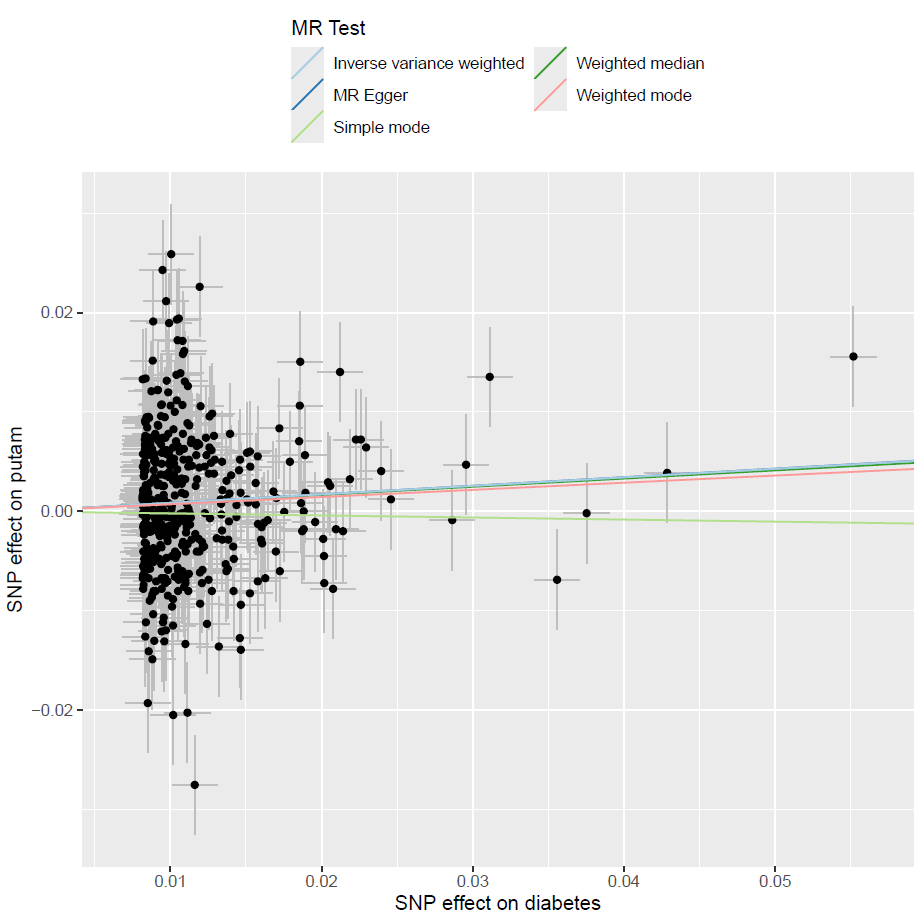
**
